# Supplementary material for: P-TRAP: a Panicle Trait Phenotyping tool
Source: BMC Plant Biol. 2013 Aug 29;13:122. doi: 10.1186/1471-2229-13-122 (PMC3848748; doi:10.1186/1471-2229-13-122)
Supplement: Additional file 7 — P-TRAP source code. The Netbeans project that contains the source code of the application. [file 1471-2229-13-122-S7.zip › javadoc.zip!/javadoc/org-basmati-workspace/help-doc.html]

API Help (WorkSpace)


JavaScript is disabled on your browser.


- Package
- Class
- Tree
- Deprecated
- Index
- Help

*org.basmati.workspace 1.0*

- Prev
- Next

- Frames
- No Frames

- All Classes

# How This API Document Is Organized

This API (Application Programming Interface) document has pages corresponding to the items in the navigation bar, described as follows.

- ## Package

  Each package has a page that contains a list of its classes and interfaces, with a summary for each. This page can contain six categories:

  - Interfaces (italic)
  - Classes
  - Enums
  - Exceptions
  - Errors
  - Annotation Types
- ## Class/Interface

  Each class, interface, nested class and nested interface has its own separate page. Each of these pages has three sections consisting of a class/interface description, summary tables, and detailed member descriptions:

  - Class inheritance diagram
  - Direct Subclasses
  - All Known Subinterfaces
  - All Known Implementing Classes
  - Class/interface declaration
  - Class/interface description
  - Nested Class Summary
  - Field Summary
  - Constructor Summary
  - Method Summary
  - Field Detail
  - Constructor Detail
  - Method Detail

  Each summary entry contains the first sentence from the detailed description for that item. The summary entries are alphabetical, while the detailed descriptions are in the order they appear in the source code. This preserves the logical groupings established by the programmer.
- ## Annotation Type

  Each annotation type has its own separate page with the following sections:

  - Annotation Type declaration
  - Annotation Type description
  - Required Element Summary
  - Optional Element Summary
  - Element Detail
- ## Enum

  Each enum has its own separate page with the following sections:

  - Enum declaration
  - Enum description
  - Enum Constant Summary
  - Enum Constant Detail
- ## Tree (Class Hierarchy)

  There is a Class Hierarchy page for all packages, plus a hierarchy for each package. Each hierarchy page contains a list of classes and a list of interfaces. The classes are organized by inheritance structure starting with `java.lang.Object`. The interfaces do not inherit from `java.lang.Object`.

  - When viewing the Overview page, clicking on "Tree" displays the hierarchy for all packages.
  - When viewing a particular package, class or interface page, clicking "Tree" displays the hierarchy for only that package.
- ## Deprecated API

  The Deprecated API page lists all of the API that have been deprecated. A deprecated API is not recommended for use, generally due to improvements, and a replacement API is usually given. Deprecated APIs may be removed in future implementations.
- ## Index

  The Index contains an alphabetic list of all classes, interfaces, constructors, methods, and fields.
- ## Prev/Next

  These links take you to the next or previous class, interface, package, or related page.
- ## Frames/No Frames

  These links show and hide the HTML frames. All pages are available with or without frames.
- ## All Classes

  The All Classes link shows all classes and interfaces except non-static nested types.
- ## Serialized Form

  Each serializable or externalizable class has a description of its serialization fields and methods. This information is of interest to re-implementors, not to developers using the API. While there is no link in the navigation bar, you can get to this information by going to any serialized class and clicking "Serialized Form" in the "See also" section of the class description.
- ## Constant Field Values

  The Constant Field Values page lists the static final fields and their values.

*This help file applies to API documentation generated using the standard doclet.*

- Package
- Class
- Tree
- Deprecated
- Index
- Help

*org.basmati.workspace 1.0*

- Prev
- Next

- Frames
- No Frames

- All Classes
